# Rare Event Approximation Between Subdistribution Hazard Ratio and Cause-specific Hazard Ratio in Survival Analysis With Competing Risks

**DOI:** 10.2188/jea.JE20240063

**Published:** 2024-12-05

**Authors:** Shiro Tanaka

**Affiliations:** Department of Clinical Biostatistics, Graduate School of Medicine, Kyoto University, Kyoto, Japan

**Keywords:** competing risk, Fine-Gray model, survival analysis, effect measure

## Abstract

**Background:**

Despite the fact that competing risks are inevitable in epidemiological and clinical studies, distinctions between the hazard ratio estimated by handling competing risks as censoring and the subditribution hazard ratio are often overlooked.

**Methods:**

We derived quantitative relationships between subdistribution hazard ratio and cause-specific hazard ratio and derive an approximate calculation method to transform the two into each other. Numerical examinations of hypothetical six scenarios and published information of a randomized clinical trial of cholesterol-lowering therapy and a registry of acute myeloid leukemia were provided.

**Results:**

General and approximate relationships under rare event assumptions between the two types of hazard ratio were given. The approximation formula is based on a survival ratio and has two possible applications. First, one can calculate a subdistribution hazard ratio from published information. Second, this formula allows sample size estimation that takes the presence of competing risks into account.

**Conclusion:**

The distinction between the two types of hazard ratio can be addressed by focusing on two quantities. One is how the event of interest and competing risk is rare, and the other is the survival ratio.

## INTRODUCTION

**Figure 1.**
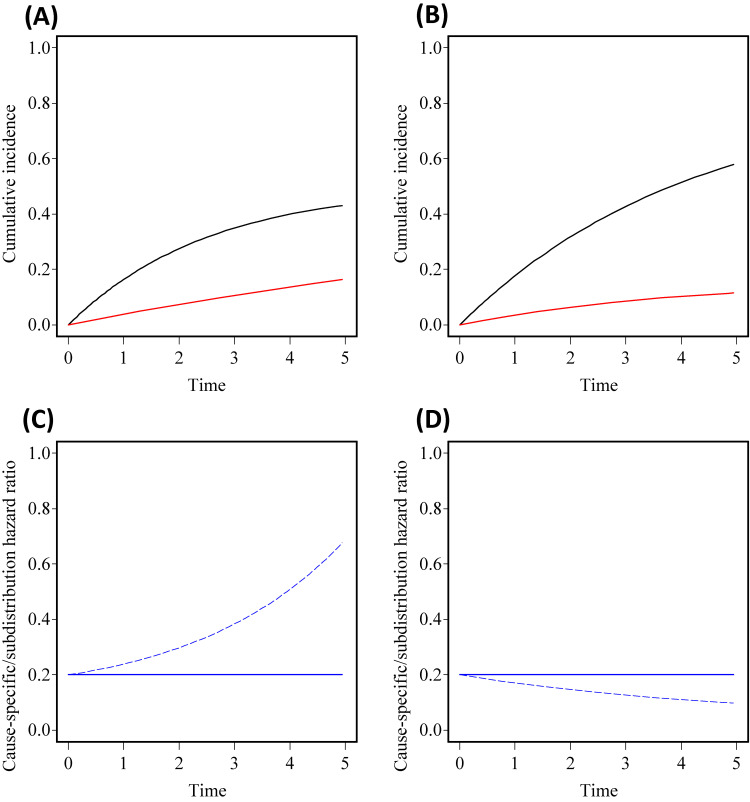
Relationships between cumulative incidence functions, cause-specific hazard ratio, and subdistribution hazard ratio. (**A**) and (**B**) show cumulative incidence functions of the treatment group (red) and the control group (black) under two scenarios with a cause-specific hazard ratio set at 0.2 and hazards of competing risk in the treatment group and the control group were set at 0.04 and 0.2 (**A**) or 0.2 and 0.04 (**B**). (**C**) and (**D**) are the corresponding cause-specific hazard ratio (solid) and the subdistribution hazard ratio (broken). All cause-specific hazards are assumed to be constant over time.

Competing risks are inevitable in epidemiological and clinical studies. Although hazard ratios estimated by handling competing risks as censoring, also termed as cause-specific hazard ratios, are routinely used, the subdistribution hazard ratio is more attractive because this measure reflects the influence of covariates on the risk of the event of interest that was actually observed. It is practically important to choose properly between these two effect measures because, the way in which covariates are associated with the cause-specific hazards may not coincide with the way suggested by the subdistribution hazard ratio of these covariates.^1^ The distinctions between cause-specific hazard ratios and subdistribution hazard ratio are as summarized in Table 1. Figure 1 illustrates how values in the two types of hazard ratios are different through numerical examples of competing risks data of two groups. Figure 1A and Figure 1B are hypothetical cumulative incidence functions of two groups. The values of hazard ratio depend on which hazard ratio is employed to summarize the cumulative incidence functions; that is, there is an increasing (left) or decreasing (right) trend in subdistribution hazard ratio while cause-specific hazard ratio is set at 0.2 (Figure 1C and Figure 1D). Dignam and Kocherginsky^2^ also demonstrated that the log-rank test and Gray test, which usually reflect each of the two types of hazard ratio, can lead to a different conclusion of the study. In this article, we clarify quantitative relationships between the subdistribution hazard ratio and the cause-specific hazard ratio and derive an approximate calculation method to transform the two into each other.

**Table 1.** Summary of distinctions between two effect measures used in competing risks analysis

	Cause-specific hazard ratio	Subdistribution hazard ratio
Quantity being compared	Cause-specific hazard function	Cumulative incidence function
	(Instantaneous risk of the event of interest at a specific time)	(Probability of experiencing the event of interest before a specific time)
Regression model	Cox regression	Fine-Gray model
	This regression model directly links the covariates to cause-specific hazard function. Cause-specific hazard ratios reflect the degree of associations.	This regression model directly links the covariates to cumulative incidence function. Subdistribution hazard ratios reflect the degree of associations.

### General relationships between hazard ratios

Consider a study comparing treated and untreated groups in a closed cohort. Participants are enrolled in the cohort at baseline and experience either the event of interest, the competing risk, or censoring during the follow-up period. For example, in a study of a causal effect on cardiovascular disease, the event of interest would be cardiovascular death and the competing risk would be death from other causes. Throughout the paper, the event of interest and competing risk are not necessarily independent, but censoring is assumed to be independent.

Our primary interest is the comparison of the risks of the event of interest between the treatment and control groups. Let *P*_1_*_E_*(*t*) and *P*_0_*_E_*(*t*) be those risks at a specific time point *t*. Let *P*_1_*_C_*(*t*) and *P*_1_*_S_*(*t*) be the probabilities of experiencing the competing risk and survival in the treatment group and *P*_0_*_C_*(*t*) and *P*_0_*_S_*(*t*) be the counterparts in the control group. These probabilities are exclusive within each group (*P*_1_*_E_*(*t*) + *P*_1_*_C_*(*t*) + *P*_1_*_S_*(*t*) = 1 and *P*_0_*_E_*(*t*) + *P*_0_*_C_*(*t*) + *P*_0_*_S_*(*t*) = 1). To compare *P*_1_*_E_*(*t*) and *P*_0_*_E_*(*t*) (or the corresponding subdistribution hazard functions), Fine and Gray^3^ introduced the subdistribution hazard ratio, denoted by *SHR*(*t*). This effect measure is usually estimated by assuming proportionality of subdistribution hazards over time^3^ but can be defined at a specific time point when one assumes other competing risks models, such as structural mean models.^4^ For the moment we consider relationships at *t*.

Suppose *SHR*(*t*) ≤ 1 throughout for simplicity. Then, the true values of *SHR*(*t*) are in general lower than or equal to the product of the cause-specific hazard ratio, denoted by *CHR*(*t*), and the survival ratio of the treatment and control groups, *P*_1_*_S_*(*t*)/*P*_0_*_S_*(*t*),

$$SHR(t)\leq \frac{P_{1S}(t)}{P_{0S}(t)}CHR(t),$$

and equality holds if and only if *SHR*(*t*) = 1 at the time point of interest, *t*. Although the fact that survival probabilities change over time is not expressed explicitly, this inequality holds at any time points. The precise treatment of time-dependence is given in eMaterial 1.

### Approximate relationships between hazard ratios under rare event assumptions

Intuitively, in a study that examines rare outcomes, no matter how one treats competing risks, the result will be approximately the same. While this intuition is correct, to address this issue accurately, we must distinguish between the event of interest and the competing risk; either is rare.

First, consider the situation where competing risks are rare (ie, *P*_1_*_C_*(*t*) < *P*_1_*_E_*(*t*) and *P*_0_*_C_*(*t*) < *P*_0_*_E_*(*t*) and *P*_1_*_C_*(*t*) and *P*_0_*_C_*(*t*) are small). In this case, the true values of *SHR*(*t*) and *CHR*(*t*) are close (eMaterial 1):

SHR(t)≈CHR(t).

On the other hand, in situations where the event of interest is rare relative to the competing risk (ie, *P*_1_*_E_*(*t*) < *P*_1_*_C_*(*t*) and *P*_0_*_E_*(*t*) < *P*_0_*_C_*(*t*) and *P*_1_*_E_*(*t*) and *P*_0_*_E_*(*t*) are small), a different approximate relationship can be established (eMaterial 1):

$$SHR(t)\approx \frac{P_{1S}(t)}{P_{0S}(t)}CHR(t).$$

In other words, the effect of competing risks when one transforms *SHR*(*t*) and *CHR*(*t*) into each other can be expressed in terms of the survival ratio.

### Sample size estimation based on the approximation formula

This formula has two possible applications. First, one can calculate a subdistribution hazard ratio from published information using this formula. For example, even if a paper reports only cause-specific hazard ratios, the survival proportions can be read off the survival curves for the all-cause event, so an estimate of subdistribution hazard ratio is usually obtained from routinely available information. Alternatively, it is possible to obtain a subdistribution hazard ratio without any approximation using the proposed formulas if the cumulative incidence functions are available (see ‘General relationships between hazard ratios’ section of eMaterial 1).

Second, this formula allows sample size estimation that takes the presence of competing risks into account. For example, one may wish to calculate sample size for comparing cumulative incidences of two groups using a Schoenfeld-type formula. Tai et al^5^ suggested several methods to derive a value of subdistribution hazard ratio required for calculation. Alternatively, the formula for total sample size *N* can be expressed without specifying the subdistribution hazard ratio,

$$N=\frac{(z_{1-\alpha /2}+z_{1-\beta }{)}^{2}}{\log(SHR{)}^{2}Pp(1-p)}\approx \frac{(z_{1-\alpha /2}+z_{1-\beta }{)}^{2}}{\log(CHR\cdot P_{1S}/P_{0S}{)}^{2}Pp(1-p)}$$

where *α*, *β* and *p* are the two-sided type I error, the one-sided type II error, and the proportion of the treatment group, and *P* = *pP*_1_*_E_* + (1 − *p*)*P*_0_*_E_* is the proportion of participants likely to experience the event of interest. Note that dependence on time is omitted in the formula; see Tai et al^5^ for discussion on proportional hazards assumptions.

### Covariate adjustment

Let *ASHR*(*t*) and *ACHR*(*t*) be a covariate-adjusted subdistribution hazard ratio and a covariate-adjusted cause-specific hazard ratio at time *t*. Then, in addition to the assumption of rare event of interest, if the covariates are neither prognostic factors nor effect modifiers or the covariate distribution is balanced between the groups, the following approximation holds because the survival ratio is a collapsible measure:

$$ASHR(t)\approx \frac{P_{1S}(t)}{P_{0S}(t)}ACHR(t).$$

This result suggests that a numerical difference between *ASHR*(*t*) and *ACHR*(*t*) would not be large in a randomized study if the crude survival proportions are not different between groups.

### Numerical examination

**Figure 2.**
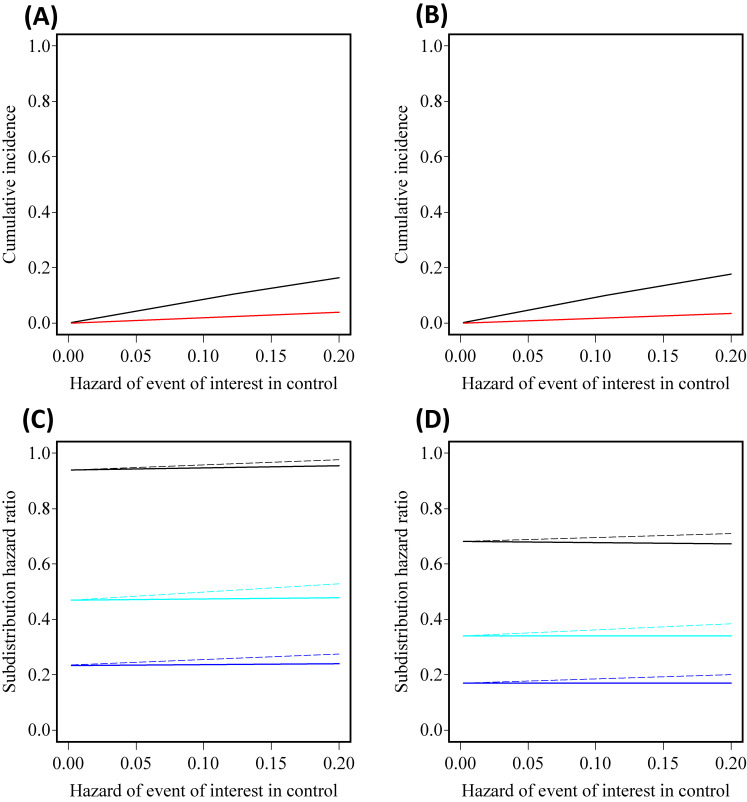
Relationships between cumulative incidence functions, cause-specific hazard ratio, subdistribution hazard ratio, and approximated subdistribution hazard ratio. (**A**) and (**B**) show associations between cumulative incidence functions at time *t* = 1 of the treatment group (red) and the control group (black) and the hazard of event of interest in the control group under two scenarios with *CHR*(*t*) = 0.2 and the hazards of competing risk in the treatment group and the control group were set at 0.04 and 0.2 (**A**) or 0.2 and 0.04 (**B**). (**C**) and (**D**) show associations between true (solid) and approximated (broken) subdistribution hazard ratios and hazard of event of interest in the control group under six scenarios. All cause-specific hazards are assumed to be constant over time. The hazards of competing risk in the treatment group and the control group were set at 0.04 and 0.2 (**C**) or 0.2 and 0.04 (**D**). The true subdistribution hazard ratios were determined based on three values of cause-specific hazard ratio (blue: *CHR*(*t*) = 0.2, cyan: *CHR*(*t*) = 0.4, black: *CHR*(*t*) = 0.8).

Figure 2 shows the accuracy of the rare event approximation under six scenarios in which all of cause-specific hazards for the event of interest and the competing risk are assumed to be constant through time. Note that subdistribution hazard functions are not proportional although the cause-specific hazard ratios are assumed to be constant. Under a scenario specified cumulative incidence functions in Figure 1A, the subdistribution hazard ratio increases from 0.2 over time, although the cause-specific hazard ratio was set at 0.2 (Figure 1B), implying that the two hazard ratios become numerically different. Figure 2A and Figure 2B show time trends up to *t* = 5, so deviation from the rare event assumptions would become more serious over time because of the increasing trends in the probabilities of developing the event and competition risk. As explained in eMaterial 1, the survival ratio, *P*_1_*_S_*(*t*)/*P*_0_*_S_*(*t*), also increases over time. Figure 2C and Figure 2D show the relationships between hazard ratios. Cause-specific hazards of competing risk in the treatment group and the control group were set at 0.04 and 0.2 (Figure 2C) or at 0.2 and 0.04 (Figure 2D); that is, the competing risk is relatively rare but its hazards are differential between the groups. The three solid lines represent three scenarios with different true values of subdistribution hazard ratio, showing that the two types of hazard ratio are quantitatively different in these scenarios. The three broken lines are approximations of the subdistribution hazard ratios obtained from survival ratios and cause-specific hazard ratios. The approximation approaches the true value as the hazard of the event of interest decreases.

### Illustration based on a randomized clinical trial of cholesterol-lowering therapy

We illustrate the use of the approximation formula through EWTOPIA75, a randomized clinical trial that revealed that cholesterol-lowering therapy with ezetimibe prevented cardiovascular events in older individuals aged ≥75 years with elevated low-density lipoprotein cholesterol.^6^ A total of 188 and 173 deaths occurred in the ezetimibe group and the control group because of the following: 29 and 45 cerebro- and cardiovascular deaths, respectively; 1 death each from the rupture of abdominal aneurysm; 120 and 98 noncerebro- and noncardiovascular deaths, respectively; and 38 and 29 deaths from malignant tumors, respectively. That trial reported that the cause-specific hazard ratios for cerebro- and cardiovascular death estimated with the conventional Cox regression was 0.656, while differences in all-cause mortality were not observed: survival proportions due to any cause at 5 years was 86% in the ezetimibe group and 87% in the control group. Based on the published results, the proposed formula yields an estimate of subdistribution hazard ratio for cerebro- and cardiovascular death of 0.648. This estimate was close to that obtained by fitting the Fine-Gray model to individual patient data (0.637).

### Illustration based on a registry of acute myeloid leukemia

Tomizawa, et al^7^ reported outcomes after hematopoietic stem cell transplantation in a nationwide registry of children and adolescent and young adult (AYA) patients with acute myeloid leukemia. An analysis of the 2,973 patients showed inferior 5-year overall survival in AYA patients (58% in children versus 54% in AYA). On the other hand, unadjusted and covariate-adjusted subdistribution hazard ratios of AYA relative to children for cumulative incidence of relapse were 0.961 and 0.999, respectively, implying that relapse in AYA patients is slightly less frequent compared with children. However, the proposed formula yields corresponding estimates of cause-specific hazard ratio of 1.032 and 1.073, while the estimates calculated using individual patient data were 1.113 and 1.123. The apparent association between age and cumulative incidence of relapse in terms of subdistribution hazard ratio is, therefore, due to the difference in overall survival.

## DISCUSSION

Limitations of our study include the following. We focused on closed cohorts with a clear origin of survival time and did not consider dynamic cohorts or complex epidemiological study designs. In these situations, the approximations may not apply. Also, even if the proportional hazards assumption holds for the subdistribution hazards, as shown in eMaterial 1, the survival ratio and the cause-specific hazard ratio may change over time. In practice, when using the approximate formulas, it is advisable to check if the proportional hazard property holds, in terms of cause-specific hazard function and subdistribution hazard function, based on individual-level data or Kaplan-Meier curves. Another issue to note is covariate adjustment that is routinely performed in observational studies. One should not expect that *ASHR*(*t*) and *ACHR*(*t*) obtained from an observational study are close even if the crude survival proportions are similar between groups, because an unadjusted comparison of survival may be subject to confounding. Finally, from a more practical point of view, the value of the survival ratio may vary depending on which point in the Kaplan-Meier curve is adopted, which may be a problem in using the approximation formula. In general terms, the right end of the Kaplan-Meier curve should not be chosen because it tends to be numerically unstable.

## SUPPLEMENTARY MATERIAL

eMaterial 1. General relationships between hazard ratios
