# Supplementary material for: Rare Event Approximation Between Subdistribution Hazard Ratio and Cause-specific Hazard Ratio in Survival Analysis With Competing Risks
Source: J Epidemiol. 2024 Dec 5;34(12):595–9. doi: 10.2188/jea.JE20240063 (PMC11564069; doi:10.2188/jea.JE20240063)
Supplement: Supplementary file 1 [file je-34-595-s001.pdf]

eMaterial 1. Relationships between subdistribution hazard ratio and cause-specific hazard ratio

General relationships between hazard ratios

Let X=0, 1 be the indicator variable for the treatment and control groups. Denote the time-to-event variable by T, the event of interest by ε=1 and the competing risk by ε=2. Cumulative incidence function and subdistribution hazard function in each group are defined as follows.

$$F_i(t|X) = \Pr(T \le t, \varepsilon = i|X)$$

$$\lambda_i(t|X) = \frac{-d \log[1 - F_i(t|X)]}{dt},$$

where i=1, 2. If a specific time point t is chosen for risk calculation, $P_{xE}(t) = F_1(t|X = x)$, $P_{xC}(t) = F_2(t|X = x)$ and $P_{xS}(t)$ is the overall survival function at t, $S(t|X = x)$. Another expression of subdistribution hazard using cause-specific hazard $h_1(t|X)$ is well-known,

$$\lambda_1(t|X) = \frac{h_1(t|X)S(t|X)}{1 - F_1(t|X)}.$$

Suppose that subdistribution hazards are proportional over time, as assumed in the Fine-Gray model. Under this assumption, subdistribution hazard ratio can be written as follows.

$$SHR(t) = \frac{\lambda_1(t|X = 1)}{\lambda_1(t|X = 0)} = \frac{S(t|X = 1)[1 - F_1(t|X = 0)]}{S(t|X = 0)[1 - F_1(t|X = 1)]}\varphi(t),$$

where we introduced a ratio of cause-specific hazards denoted as $\varphi(t) = h_1(t|X = 1)/h_1(t|X = 0)$. This equation represents a fundamental relationship between subdistribution hazard ratio and cause-specific hazard ratio. Obviously, $\varphi(t)$ in this equation is a function of time, showing that, in general, the proportional hazards assumption in terms of subditribution hazard and the proportional hazards assumption in terms of cause-specific hazard do not hold simultaneously. Specifically, $SHR(t) = \varphi(t) = 1$ if and only if both cause-specific hazards of the event of interest and the competing risk are equal between the two groups. Otherwise, even if $SHR(t)$=1, the cause-specific hazards of the two groups are different.

It is shown that these two measures are linked through all-cause odds ratio (AOR), all-cause risk ratio (ARR), subdistribution odds ratio (SOR) and subdistribution risk ratio (SRR):

$$SHR(t) = \frac{ARR}{AOR}\frac{SOR}{SRR}\varphi(t),$$

where

$$AOR(t) = \frac{[1 - S(t|X = 1)]/S(t|X = 1)}{[1 - S(t|X = 0)]/S(t|X = 0)}$$

$$ARR(t) = \frac{1 - S(t|X = 1)}{1 - S(t|X = 0)}$$

$$SOR(t) = \frac{F_1(t|X=1)/[1-F_1(t|X=1)]}{F_1(t|X=0)/[1-F_1(t|X=0)]}$$

$$SRR(t) = \frac{F_1(t|X=1)}{F_1(t|X=0)}.$$

Suppose *SHR*≤1. Then, $F_1(t|X=1) \le F_1(t|X=0)$ and therefore $SOR(t) \le SRR(t)$. This means

$$SHR(t) = \frac{ARR(t)}{AOR(t)}\frac{SOR(t)}{SRR(t)}\varphi(t) \le \frac{ARR(t)}{AOR(t)}\varphi(t) = \frac{S(t|X=1)}{S(t|X=0)}\varphi(t).$$

Relationships with an all-cause hazard ratio,

$$\theta(t) = \frac{h_1(t|X=1) + h_2(t|X=1)}{h_1(t|X=0) + h_2(t|X=0)},$$

may worth presenting. If the cause-specific hazard ratio of competing risk is larger than that of event of interest, that is, if $\varphi(t) < h_2(t|X=1)/h_2(t|X=0)$, then the following inequalities also hold.

$$\varphi(t) < \theta(t)$$

$$SHR(t) < \frac{S(t|X=1)}{S(t|X=0)}\theta(t)$$

Otherwise, $\theta(t) \le \varphi(t)$.

Approximate relationships between hazard ratios when only competing risk is rare

In this case, $h_2(t|X)$ is small. Thus, overall survival function can be approximated by cumulative incidence function.

$$S(t|X) = 1 - \int_0^t h_1(s|X)S(s|X)ds - \int_0^t h_2(s|X)S(s|X)ds \approx 1 - F_1(t|X)$$

This means that $AOR(t) \approx SOR(t)$ and $ARR(t) \approx SRR(t)$. Combining this with the equality linking $SHR(t)$ and $\varphi_1(t)$ yields $SHR(t) \approx \varphi(t)$.

Approximate relationships when only event of interest is rare

Because $F_1(t|X)$ is small in this case, $SRR(t) \approx SOR(t)$. This gives the following relationships.

$$SHR(t) = \frac{ARR(t)}{AOR(t)}\frac{SOR(t)}{SRR(t)}\varphi(t) \approx \frac{ARR(t)}{AOR(t)}\varphi(t) = \frac{S(t|X=1)}{S(t|X=0)}\varphi(t).$$
